# Guideline review: 2025 British Society of Gastroenterology guidelines on inflammatory bowel disease in adults – Part 1: Crohn’s disease

**DOI:** 10.1136/flgastro-2025-103425

**Published:** 2025-11-25

**Authors:** Thomas D Butler, Jonathan P Segal

**Affiliations:** 1 Division of Diabetes, Endocrinology and Gastroenterology, Faculty of Biology, Medicine and Health, The University of Manchester, Manchester, M13 9PT, UK; 2 Northern Care Alliance NHS Foundation Trust, Salford, Manchester, UK; 3 Department of Gastroenterology, Royal Melbourne Hospital, Parkville, Victoria, Australia; 4 Department of Medicine, University of Melbourne, Parkville, Victoria, Australia

## Abstract

This guideline review summarises the key recommendations for the diagnosis and management of Crohn’s disease from the updated 2025 British Society of Gastroenterology guidelines on inflammatory bowel disease in adults.

## Introduction

In Crohn’s disease (CD), chronic inflammation can affect any part of the gastrointestinal tract, leading to a range of clinical presentations that frequently require multiple investigations to reach a diagnosis. Intestinal and extraintestinal manifestations of CD can have a major impact on quality of life. The transmural nature of inflammation can lead to strictures, fistulae, abscesses and perianal disease, presenting challenges for management with medical and surgical therapy. Recent reports from IBD UK (2024) ^1^ and NCEPOD (2023) ^2^ highlight the importance of striving to improve services to care for people living with IBD. Since the 2019 British Society of Gastroenterology (BSG) guidelines, there have been significant breakthroughs in the management of CD, with the exciting prospect of advancing IBD care. This article summarises the key recommendations for the management of CD included in the 2025 BSG IBD Guidelines^3^.

## Methodology

A Delphi process prospectively identified and prioritised key thematic questions for this guideline update. Guideline recommendations adhere to GRADE methodology, as described in the guideline protocol ^4^. An annotated explanation of GRADE language is included within the guideline to aid readers.

### Diagnosis and monitoring of Crohn’s disease

The Montreal classification is still recommended to describe CD phenotypes. The diagnosis of CD is supported by blood tests, faecal calprotectin, cross-sectional imaging, endoscopy and histology. The combination of required investigations is determined by clinical presentation and findings. Investigation of small bowel inflammation often requires cross-sectional imaging, and guidelines suggest MRI enterography as first line to limit exposure to ionising radiation. There are benefits and limitations with all modalities including CT, MRI and intestinal ultrasound (IUS) and an individualised approach is required. Faecal calprotectin is an important non-invasive monitoring tool in CD patients.

These guidelines highlight the potential utility of IUS, supported by a growing evidence base. The METRIC trial demonstrated that IUS was a sensitive and specific test for detecting small bowel inflammation, albeit statistically inferior to MRI ^5^. The TRUST study showed that IUS could successfully monitor response to treatment following a flare ^6^. Going forward, IUS will likely play a role as a non-invasive, patient-friendly alternative to endoscopic examination, and the IBD community should continue to advocate for local resource allocation to integrate IUS into IBD units across the UK.

During assessment of CD with colonoscopy, ileal intubation and examination is important to visualise the commonly affected terminal ileum. In patients with prior ileocolonic resection and anastomosis, the Rutgeerts’ score remains the most common method of describing endoscopic recurrence. Small bowel capsule endoscopy is suggested when CD is suspected despite normal initial investigations, but the risk of capsule retention, particularly with stricturing CD often necessitates prior screening with a patency capsule. Upper gastrointestinal CD is less common and gastroscopy is only required in the presence of upper gastrointestinal symptoms.

### Management of Crohn’s disease

In IBD, the approach to treatment includes induction and maintenance of remission. Ileal-release budesonide is a useful treatment for mild CD, sparing the more potent corticosteroids for induction of remission in moderate to severe CD. However, the guidelines highlight the importance of avoiding lengthened or repeat corticosteroid courses, due to the significant side effect profile, and growing evidence to support early ‘top down’ advanced therapy initiation.

### A ‘top down’ approach to treatment of moderate to severe Crohn’s disease

Across immune-mediated inflammatory disease, there is a move towards earlier treatment with advanced therapies to reduce long-term harms of uncontrolled inflammation. Most recently in CD, the PROFILE trial recruited participants with clinical, biochemical and endoscopic evidence of moderate to severe inflammation, and demonstrated that early top down initiation of infliximab in combination with an immunomodulator was superior to ‘step up’ therapy at increasing surgery-free and steroid-free remission at week 48 (ref ^7^). Infliximab should be initiated early in patients presenting with moderate to severe CD. It is likely, but not yet evidenced that this pivotal finding extrapolates to other advanced therapies. The role and utility of top down therapy in mild CD is unknown.

### Immunomodulator monotherapy

The guidelines do not suggest immunomodulator monotherapy with either methotrexate or purine analogues for the induction or maintenance of moderate to severe CD. This is a move away from the 2019 recommendations and aligns with the emergence of a top down approach to management. However, withdrawal of immunomodulator monotherapy is associated with relapse in 1/3 of patients, and those already established on immunomodulator monotherapy should not have their treatment routinely stopped prior to counselling of the available options.

### Advanced therapy

In 2019, available advanced therapies for CD included infliximab, adalimumab, vedolizumab and ustekinumab. This guideline removes vedolizumab (see below) and adds upadacitinib and risankizumab as advanced therapies suggested for induction and maintenance of remission in moderate to severe CD (Table 1). Of note, mirikizumab was not licensed for use in CD at the time of guideline publication.

Anti-TNF therapy remains an important, and often initial, advanced therapy in patients with moderate to severe CD. Both infliximab and adalimumab are recommended to be initiated in combination with purine analogues to reduce immunogenicity. Routine withdrawal of advanced therapy is not suggested, due to high odds of relapse in 1/3 patients within 1-2 years, even when prolonged mucosal healing is achieved. Several factors influence drug withdrawal and decisions should be made jointly with the patient, with close follow up monitoring for relapse.

### Therapeutic drug monitoring

Therapeutic drug monitoring (TDM) of purine analogues is well established, to ensure safe, therapeutic levels. In combination with anti-TNF therapy, purine analogues predominantly play a role in reducing immunogenicity and thus TDM levels are less well defined. The guidelines suggest accepting relatively lower 6-TGN levels >125 pmol/8x10^8^ RBCs, with dose increases as required to reduce emerging immunogenicity. MMP levels <5700 pmol/8x10^8^ RBCs reduce risk of hepatotoxicity.

TDM of anti-TNF therapies, including measurement of trough serum infliximab concentration and serum anti-drug antibodies, is becoming more widespread, supported by multiple studies demonstrating positive correlation between higher trough levels and clinical outcomes. In daily practice, TDM may have a role in situations such as primary non-response to anti-TNF therapy, to inform dose escalation. In secondary loss of response, TDM could determine presence of anti-drug antibodies and inform therapy switching. However, optimal target TDM levels are yet to be defined, influenced by several factors including disease activity, assay manufacturer and subcutaneous versus intravenous route of administration for infliximab. In addition, a proactive approach to trough level measurement, where TDM is performed at predefined intervals during anti-TNF initiation, has not consistently demonstrated benefit over standard of care.

The guidelines present a good practice statement that TDM can support dose escalation, which may lead to better clinical outcomes. Immunomodulator therapy should be started concomitantly with anti-TNF therapy to minimise the risk of immunogenicity. It is unlikely that patients with high titres of anti-drug antibodies benefit from dose escalation and advanced therapy switching should be considered. There is scope for further research to define the best approach to TDM of anti-TNF therapy. There is currently no role for TDM in non-anti-TNF advanced therapies.

### Vedolizumab is not suggested for induction and maintenance of remission in patients with moderate to severe Crohn’s disease

Notably, and in a departure from previous guidelines, vedolizumab is no longer a suggested therapy for moderate to severe CD. This GRADE statement follows a Cochrane review including 1025 participants across four randomised controlled trials (RCT) for induction and three RCTs with 895 participants for maintenance, which demonstrated trivial and small magnitudes of effect for induction and maintenance of remission in CD, respectively. As noted by the guideline authors, non-randomised trials are not included in evidence synthesis for the GRADE statements. Thus, data were not included from LOVE-CD, a prospective, but non-randomised study demonstrating a third of participants treated with vedolizumab after anti-TNF failure achieved clinical and endoscopic remission at 52 weeks ^8^. Vedolizumab likely retains a role in selected populations with moderate to severe CD, but patients should be informed that their odds of response are lower than other therapies and MDT agreement for vedolizumab use is advised.

### Surgery in Crohn’s disease

There is currently insufficient evidence to suggest exclusive enteral nutrition (EEN) for treatment of CD routinely, but guidelines do provide a practical guide to using EEN to induce remission in adults with CD over a 6-8 week period, using expert consensus. Pre-operative EEN can also be considered with the aim of reducing surgical risk, and is the subject of ongoing research.

Prior research has demonstrated equipoise between medical and surgical intervention for limited ileocaecal CD ^9^ and ileocaecal resection remains a valid therapy for limited CD, and should be considered as an early treatment option in patients alongside discussions to initiate or continue advanced medical therapies. However, as highlighted in the NCEPOD report ^2^, ongoing delays to timely, elective, benign IBD surgery across the country should be taken into account.

### Maintenance of remission post-ileocaecal resection

Following ileocaecal resection with primary anastomosis, early initiation of advanced therapy to maintain remission is offered to high risk patients, such as those with penetrating disease, multiple surgical resections, perianal disease and active smoker status. Trials included in the network meta-analysis (NMA) for this guideline recruited participants within 3 months of surgery and rates of relapse in placebo arm was significant, which challenges the requirement to wait for 6 month endoscopic anastomotic reassessment to guide maintenance treatment. The guideline now suggests that all post-surgical patients are given the opportunity to receive early advanced therapy, regardless of high risk status. infliximab, adalimumab or vedolizumab should be considered initially. Of note, this vedolizumab statement, compared with the removal of vedolizumab from the CD treatment options highlights the heterogeneity of CD, the oft contradictory nature of available evidence and the need for ongoing, robust, high-quality trials across IBD.

No other treatments are suggested for post-surgical maintenance of remission including probiotics, curcumin and 5-ASA therapy. This also includes antibiotics, which did not reach pooled significance in the NMA. Expert consensus does support the use of a 3-month course of a nitroimidazole such as metronidazole, based on limited available evidence of reduced clinical and endoscopic recurrence. However, the unpalatability and side effect profile of metronidazole often leads to poor compliance.

### Future directions

IBD trial design must evolve to avoid harms of receiving placebo ^10^, with consideration for novel approaches such as platform studies and head-to-head studies to inform comparative efficacy and identify factors influencing individualised treatment choices. GRADE methodology prioritises randomised controlled trials for statements of efficacy. To ensure future guidelines continue to present high quality statements, the IBD community, including patients must continue to identify priority questions and undertake collaborative high-quality research. The guidelines provide a wealth of opportunities for local service development and quality improvement.

## Conclusions

The management of CD remains a prominent challenge for clinicians. The *BSG guidelines on IBD in adults* provide an important and wide-ranging update, including guidance on the diagnosis and monitoring of CD, and specifically including methodologically rigorous guidance on the timing and initiation of available therapies to manage CD. Personalised IBD care has not yet materialised, but focus on a patient-centred shared decision-making approach through this guideline will aid clinicians in finding the correct management strategy for each person with IBD.

## Figures and Tables

**Table 1:** A comparison of treatment recommendations for moderate-to-severe Crohn’s disease between 2019 and current 2025 guidelines. Box colours: green, recommendation for use; orange, no recommendation for use; grey, not available for use in 2019. Abbreviations: ASA, aminosalicylate; TNF, tumour necrosis factor; JAK, janus kinase; IL, interleukin

Drug class	Drug	2019 guideline	2025 guideline	Notes
Aminosalicylate	5-ASA			
Steroid	Corticosteroids			Caution with repeated or lengthened courses
Immunomodulator	Purine analogue monotherapy			
	Methotrexate monotherapy			
Anti-TNF	Infliximab			Combination therapy with immunomodulator recommended at anti-TNF initiation
	Adalimumab			
JAK inhibitor	Upadacitinib			
Anti-IL-23	Risankizumab			
Anti-IL-12/23	Ustekinumab			
Anti-integrin	Vedolizumab			Guidelines suggest use for post-surgical maintenance of remission

## What is already known on this topic

Inflammatory bowel disease (IBD), consisting of Crohn’s disease and ulcerative colitis, affects a range of people with significant impact on quality of life. IBD diagnosis and management can be challenging.

## What this study adds

The 2025 BSG guidelines on IBD in adults provides an update from the 2019 guidelines, reflecting recent advancements, particularly relating to novel therapeutic agents.

## How this study might affect research, practice or policy

This guideline provides clinicians with a comprehensive overview of the approach to diagnosis and management of people with IBD. This review covers management of Crohn’s disease.
